# Effects of DPP4 Inhibitors as Neuroprotective Drug on Cognitive Impairment in Patients with Type 2 Diabetes Mellitus: A Meta-Analysis and Systematic Review

**DOI:** 10.1155/2024/9294113

**Published:** 2024-02-13

**Authors:** Yuting Yuan, Yue Zhang, Min Lei, Xiying Guo, Xiaosong Yang, Changhan Ouyang, Chao Liu, Qingjie Chen

**Affiliations:** ^1^School of Pharmacy, Xianning Medical College, Hubei University of Science and Technology, Xianning, Hubei, China; ^2^Hubei Key Laboratory of Diabetes and Angiopathy, Medicine Research Institute, Xianning Medical College, Hubei University of Science and Technology, Xianning, Hubei, China

## Abstract

**Purpose:**

Type 2 diabetes mellitus is considered as one of the risk factors for cognitive impairment. DPP4 inhibitors are effective drugs for the treatment of type 2 diabetes mellitus. However, the relationship between DPP4 inhibitors and cognitive dysfunction remains unclear. Therefore, we used a meta-analysis to determine the association between DPP4 inhibitors and cognitive impairment in type 2 diabetes mellitus.

**Methods:**

We systematically searched PubMed, CNKI, and the Cochrane Library at the time of establishment, 2022, and then made inclusion criteria and screened strategies to identify studies with more precise correlations.

**Results:**

We included 10 studies with 5,583 participants. The data showed that DPP4 inhibitors significantly reduced the incidence rate of cognitive impairment in type 2 diabetes mellitus (SMD: 0.99; 95% CI [0.59, 1.38]). Furthermore, there was a linear correlation found between cognitive impairment in type 2 diabetes mellitus and fasting blood glucose, 2-hour postprandial blood glucose, and glycosylated hemoglobin. DPP4 inhibitors decreased fasting blood glucose (FPG) (SMD: 0.52; 95% CI [−0.68, −0.37]), blood glucose (2hPPG) at 2 hours after the meal (SMD: 0.82; 95% CI, [−1.2, −0.43]), and HbA1c (SMD: 0.34; 95% CI [−0.48, −0.21]). All data were statistically significant (*P* < 0.0001). Furthermore, we conducted subgroup analyses of the following measures at various treatment durations and ages: cognitive scores, fasting blood glucose, glycosylated hemoglobin, and two-hour postprandial blood glucose.

**Conclusion:**

DPP4 inhibitors significantly improved type 2 diabetic mellitus individuals' cognitive impairment and reduced fasting blood glucose, 2-hour postprandial blood glucose, and glycosylated hemoglobin. Subgroup analysis showed that people aged 60 to 70 years had better treatment effects at 0–180 days. This trial is registered with CRD42023399473.

## 1. Introduction

A risk factor for cognitive dysfunction, such as dementia, mild cognitive impairment, and cognitive decline, is type 2 diabetes mellitus (T2D) [1]. Cognitive dysfunction is deemed to be the presence of a number of cognitive disabilities that result in occupational dysfunction [2]. Several researchers [3] have reported that people with type 2 diabetes mellitus generally suffer from cognitive dysfunction. The intimate relationship between these two conditions has been proved many times as many as 60% of T2D patients have cognitive dysfunction [4]. In addition, the quality of life of T2D patients is rapidly declining due to an increase in cognitive impairment disorders. The lack of effective treatments for cognitive impairment in diabetic patients necessitates the search for effective medications.

In previous studies, increased activity of dipeptidyl peptidase-4 inhibitors (DPP4) was independently associated with mild cognitive impairment (MCI) in elderly patients with T2D [5]. DPP-4, an intestinal glucagon-like peptide-1 (GLP-1) degrading enzyme, can block DPP-4 to reduce GLP-1 degradation, prolong its GLP-1 effect when used, and lead to insulin activity, thus reducing blood sugar [6]. In addition, GLP-1 is thought to be a neurotrophic factor that prevents neurodegeneration, possibly through long-term enhancement, enhances synapse growth, and promotes synapse formation in a manner similar to nerve growth factor [7]. DPP4 inhibitors can effectively prevent the degradation of glucagon-like peptide-1 and gastric inhibitory peptide (GIP). Thus, the half-life of these enteric insulins prolongs neuronal lifespan [8, 9]. Such mechanisms include activation of the mTOR pathway and tau hyperphosphorylation, thus inhibiting liver gluconeogenesis, reducing insulin resistance, increasing insulin sensitivity, and inhibiting inflammation [10–12].

In a Danish real-world study, DPP4 inhibitors showed better cognitive outcomes compared to sulfonylureas [13]. The results of another retrospective study also showed that DPP4 inhibitors were protective against cognitive impairment compared with metformin [14]. It seems that these studies are all about the beneficial effects of DPP4 inhibitors. However, other studies have suggested that DPP4 inhibitors may be associated with an increased risk of cognitive impairment in T2D patients. DPP4 is widely used in diabetes treatment; the additional beneficial role of drugs on cognitive function has been recommended. We conducted a meta-analysis to evaluate cognitive outcomes in T2D patients treated with DPP4 inhibitors.

## 2. Material and Methods

### 2.1. Data Source

**Figure 1:**
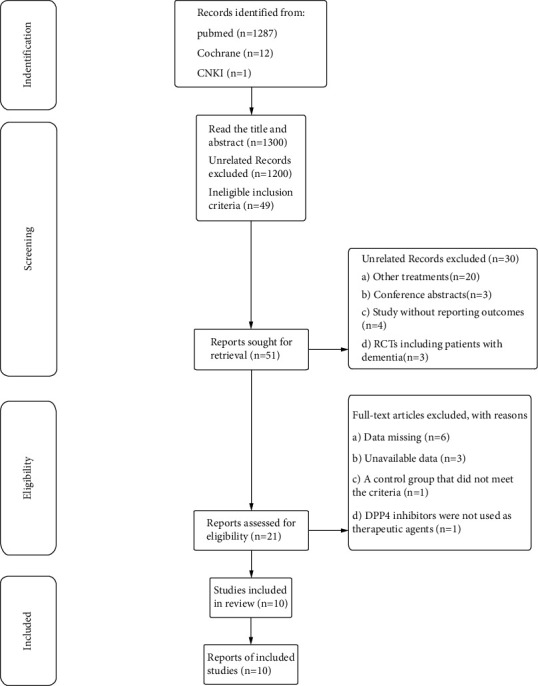
Flowchart of study selection for the meta-analysis.

The results of this systematic review and meta-analysis have been written based on the PRISMA checklist (Supplementary PRISMA checklist). The literature search was summarized by two researchers (Yuting Yuan and Qingjie Chen) who searched four databases through medical keywords (MeSH) and keywords, respectively. The original PubMed, Cochrane, and CNKI randomized controlled trials (RCTS) of DPP4 inhibitors on cognitive impairment in T2D were searched (2022) without language restrictions. The results will be examined by the third team part (Yue Zhang). After the review, the two participants included the literature that made the inclusion criteria, respectively, by reading the title and abstract. In the event of any disagreement, the two parties shall negotiate to decide. If the discussion fails to resolve the disagreement, the opinion of a third party will be adopted. The meta-analysis was proceeding according to the Cochrane Handbook of Systematic Reviews of Interventions [15]. The researchers reviewed and analyzed all the collected literature (Figure 1).

### 2.2. Search Strategy

We systematically searched PubMed, CNKI, and the Cochrane Library at the time of establishment, 2022, and the search strategy is ((((DPP-4 Inhibitor) OR (DPP-IV Inhibitor) OR (Dipeptidyl Peptidase 4 Inhibitor)) AND (diabetes Cognitive Dysfunctions)) OR (diabetes cognitive Impairment)) OR (diabetes mild cognitive Impairments) Sort by: Most Recent((“dipeptidyl-peptidase iv inhibitors”[All Fields] OR “dipeptidyl-peptidase iv inhibitors”[MeSH Terms] OR (“dipeptidyl-peptidase”[All Fields] AND “iv”[All Fields] AND “inhibitors”[All Fields]) OR “dipeptidyl-peptidase iv inhibitors”[All Fields] OR “dpp 4 inhibitor”[All Fields]) OR (“dipeptidyl-peptidase iv inhibitors”[All Fields] OR “dipeptidyl-peptidase iv inhibitors”[MeSH Terms] OR (“dipeptidyl-peptidase”[All Fields] AND “iv”[All Fields] AND “inhibitors”[All Fields]) OR “dipeptidyl-peptidase iv inhibitors”[All Fields] OR (“dpp”[All Fields] AND “iv”[All Fields] AND “inhibitor”[All Fields]) OR “dpp iv inhibitor”[All Fields]) OR (“dipeptidyl-peptidase iv inhibitors”[All Fields] OR “dipeptidyl-peptidase iv inhibitors”[MeSH Terms] OR (“dipeptidyl-peptidase”[All Fields] AND “iv”[All Fields] AND “inhibitors”[All Fields]) OR “dipeptidyl-peptidase iv inhibitors”[All Fields] OR “dipeptidyl-peptidase 4 inhibitor”[All Fields])) AND (((“diabetes mellitus”[MeSH Terms] OR (“diabetes”[All Fields] AND “mellitus”[All Fields]) OR “diabetes mellitus”[All Fields] OR “diabetes”[All Fields] OR “diabetes insipidus”[MeSH Terms] OR (“diabetes”[All Fields] AND “insipidus”[All Fields]) OR “diabetes insipidus”[All Fields]) AND (“cognitive dysfunction”[MeSH Terms] OR (“cognitive”[All Fields] AND “dysfunction”[All Fields]) OR “cognitive dysfunction”[All Fields] OR (“cognitive”[All Fields] AND “impairments”[All Fields]) OR “cognitive impairments”[All Fields])) OR ((“diabetes mellitus”[MeSH Terms] OR (“diabetes”[All Fields] AND “mellitus”[All Fields]) OR “diabetes mellitus”[All Fields] OR “diabetes”[All Fields] OR “diabetes insipidus”[MeSH Terms] OR (“diabetes”[All Fields] AND “insipidus”[All Fields]) OR “diabetes insipidus”[All Fields]) AND (“cognitive dysfunction”[MeSH Terms] OR (“cognitive”[All Fields] AND “dysfunction”[All Fields]) OR “cognitive dysfunction”[All Fields] OR (“cognitive”[All Fields] AND “dysfunctions”[All Fields]) OR “cognitive dysfunctions”[All Fields])) OR (“cognitive dysfunction”[MeSH Terms] OR (“cognitive”[All Fields] AND “dysfunction”[All Fields]) OR “cognitive dysfunction”[All Fields] OR (“mental”[All Fields] AND “deterioration”[All Fields]) OR “mental deterioration”[All Fields]))

Cochrane Library trials matching diabetes in Title Abstract Keyword AND DPP-4 Inhibitor or Dipeptidyl Peptidase IV Inhibitors or DPP-IV Inhibitors in Title Abstract Keyword AND Cognitive Dysfunctions or Cognitive Impairments or Mental Deterioration in Title Abstract Keyword - (Word variations have been searched).

A total of 1287 articles were retrieved from PubMed, 1 from CNKI, and 12 from Cochrane Library.

### 2.3. Data Extraction

Inclusion and exclusion criteria were drawn up based on PICOS.

The inclusion criteria are as follows:

1. Published reports on the effects of DPP4 inhibitors (Sitagliptin, Vildagliptin, Linagliptin, Alogliptin, and Saxagliptin) on people with diabetic cognitive impairment
2. The study design must be an RCTS with placebo or parallel controls
3. Patients must conform to the definition of diabetes and have a positive pathological examination or OGTT test (fasting blood glucose ≥7.0 mmol/l or blood glucose ≥11.1 mmol/l 2 hours after meal)
4. The patient must have cognitive impairment caused by the underlying disease of diabetes and have the same cognitive scoring tools between the experimental group and the control group
5. The drug in the experimental group must be a DPP4 inhibitor

The exclusion criteria are as follows:

1. The deviation of research data is obvious, and the reliability is low
2. Patients with diabetes or cognitive impairment were not involved
3. There was no DPP4 inhibitor in the experimental group
4. Literature without data

### 2.4. Data Quality Assessment

The following data were recorded for each study: first author, year of publication, participants (gender, age, course of disease, sample size, and history of cognitive impairment), study design (observational cohort or randomized controlled study), years of follow-up, dosage and risk (HR), and 95% confidence interval (CI). According to the Cochrane Handbook for Systematic Evaluation of Intervention [15], the quality of selected studies was evaluated in six aspects: assignment hiding, random sequence generation, outcome evaluation blindness, participant and personnel blindness, incomplete outcome data, and selective reporting. These biases will be independently assessed by 2 review authors for each included study. Among them, the definition of allocation hiding is the selective bias caused by the imperfect follow-up allocation scheme, and the definition of selective reporting is the reporting bias caused by the selective reporting results. The literature we included included one risk on the allocation of hidden risks and six risks due to incomplete other data in the selective report. The Cochrane bias risk assessment tool will be used to mark each bias as “yes” (low bias risk), “no” (high bias risk), or “unclear” (uncertain bias risk). Any disagreement will be discussed with the third review writer. If there is any disagreement, please contact the author for a request.

### 2.5. Data Analysis

In this study, basic information about the relevant literature, baseline information and experimental results, year of journal publication, authors' details, duration of the study, study population, duration of follow-up, age at baseline including title of the article, baseline weight, gender distribution, duration of diabetes mellitus, definition of the endpoints, and determination of Montreal Cognitive Assessment Score [16] and the results of the study were extracted. Ratio and 95% confidence intervals (CI) will be calculated to assess the relationship between DPP-4 inhibitors and cognitive impairment. In general, we compared the baseline data before and after medication, extracted the mean, standard deviation, and sample size (N) from the literature, and counted the mean (Mean) and standard deviation (SD) values difference between baseline and endpoint. Considering the differences in experimental design and measurement units, differences in the data were eliminated by standardized mean difference (SMD) [17]. We used the random-effects model or fixed-effects model in RevMan5.4 software (the selection criterion was to test the heterogeneity of the high heterogeneity derandomization effect model with *I*^2^ > 75% and that of the low heterogeneity defixation effect model with *I*^2^ < 25%) to count the SMD and 95% CI. We used forest plots to analyse effect sizes. *P* < 0.05 was considered statistically significant.

## 3. Results

### 3.1. Search Result

Twenty-one studies were initially identified in the PubMed database (*n* = 1287), CNKI1 (*n* = 1), and Cochrane 12 (*n* = 12) (Figure 1). Twenty-one articles were manually retrieved for full-text review, and nondata, different life intervention strategies, nonblank, and nonrandomized controlled trials were excluded. The remaining 10 articles meet the requirements. Of the 10 included trials, 2 were controlled by placebo and 8 were controlled by other hypoglycemic agents (Supplementary Table 1) [13, 14, 18–25].

### 3.2. Data Selection

When evaluating the effect of DPP4 inhibitors on cognitive impairment in T2D, we selected the Montreal Score (MoCA) or the MMSE as an indicator to improve cognitive impairment [15]. In addition, fasting blood glucose, 2-hour post meal blood glucose, and glycosylated hemoglobin are associated with cognitive impairment. According to previous studies, the TyG index (calculated as in (fasting triglyceride [mg/dL] × fasting blood glucose [mg/dL]/2)) is associated with the risk of cognitive decline in diabetic patients [26]. People with prediabetes and diabetes who had high levels of glycosylated hemoglobin (HbA1c) compared to normal blood sugar showed significant cognitive decline over 10 years. When HbA1c is increased by 1 mmol/ml, the *Z*-score of global cognition (−0.0009 SD/year), the Z-score of memory (−0.0005 SD/year), and the *Z*-score of executive function (−0.0008 SD/year) all decrease [27]. The study found a linear correlation between cognitive dysfunction and glycosylated hemoglobin levels. Thus, effectively reducing fasting blood glucose (FPG), 2-hour postprandial blood glucose (2hPPG), and glycosylated hemoglobin (HbA1C) can also improve cognitive impairment.

### 3.3. Risk of Bias Assessment

**Figure 2:**
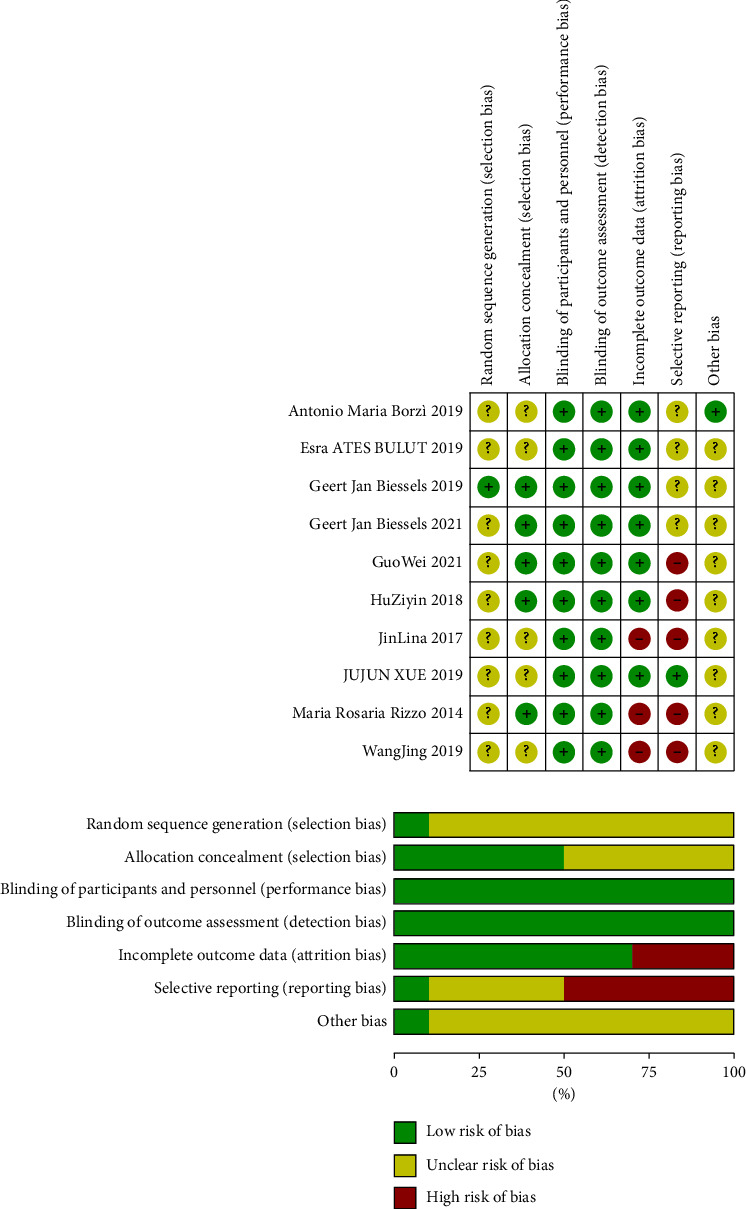
Assessment of risk of bias. As shown in the figure, the risk of bias was evaluated in six dimensions. Low risk of bias was represented by green, unclear risk of bias was represented by yellow, and high risk of bias was represented by red.

All 10 included trials were declared randomized, and 10 used a double-blind study design. In the 10 trials, placebo-controlled was 2, and other hypoglycemic agents controlled was 8 (Figure 2).

### 3.4. Relationship between DPP4i and Cognitive Score

**Figure 3:**
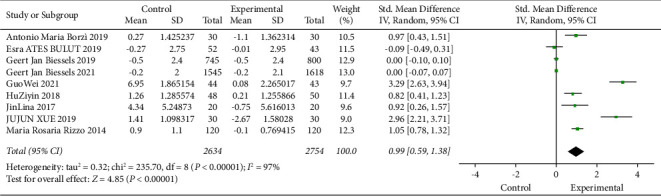
Meta-analysis of the impact of DPP4 inhibitors on cognitive impairment. Forest map displaying variations in MMSE with diabetes treated or not with DPP4i in 9 trials (*n* = 5388). Black squares indicate mean differences, horizontal lines through black squares indicate 95% CI, and green diamonds indicate pooled effect sizes shown using a random-effects Hedges model.

Figure 3 illustrates the effect of DPP4 inhibitors on cognitive impairment in patients with T2D. Random-effects models were used, including 2634 in the control group and 2754 in the experimental group. After standardization, the MMSE score of the test group increased by 0.99 (95% [0.59, 1.38]), and *I*^2^ value was 97%, *P* < 0.00001 (statistically significant), showing high heterogeneity. The data suggest that DPP4 inhibitors can improve cognitive impairment in patients.

### 3.5. Relationship between DPP4i and Fasting Blood Glucose

**Figure 4:**
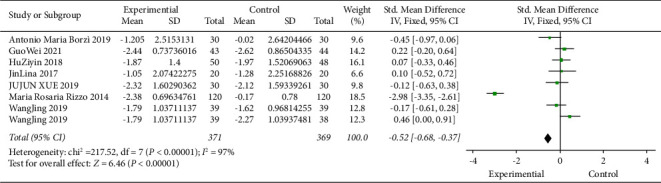
Meta-analysis of the impact of DPP4 inhibitors on fasting blood glucose. Forest map showing the difference in fasting blood glucose variations with diabetes treated or not treated with DPP4i in 8 trials (*n* = 740). Black squares indicate mean differences, horizontal lines through black squares indicate 95% CI, and green diamonds indicate pooled effect sizes shown using a fixed-effect Hedges model.

Previous studies have shown that patients with elevated serum triglycerides (TG) and blood sugar levels have a greater risk of cognitive impairment than those with lower levels. One index, TyG index (calculated as in (fasting triglyceride [mg/dL] × fasting blood glucose [mg/dL]/2)), is associated with the risk of cognitive decline in patients with diabetes.  Figure 4 illustrates the efficacy of DPP4 inhibitors on fasting glucose. A fixed-effect model was used in 371 subjects in the experimental group and 369 subjects in the control group. After standardization, compared with the control group, fasting blood glucose in the experimental group was reduced by 0.52 (95% CI [−0.68, −0.37]), *I*^2^ was 97% (*P* < 0.05). This figure indicates that DPP4 inhibitors effectively reduce fasting blood glucose and thus effectively improve cognitive impairment in diabetic patients.

### 3.6. Relationship between DPP4i and Glycosylated Hemoglobin

**Figure 5:**
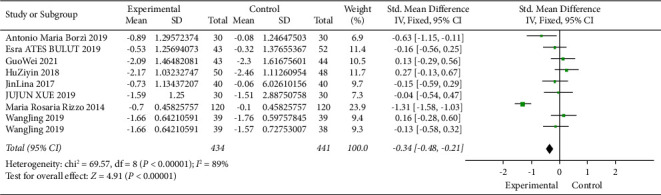
Meta-analysis of the impact of DPP4 inhibitors on glycosylated hemoglobin. Forest map showing the difference in glycosylated hemoglobin variations with diabetes treated or not treated with DPP4i in 9 trials (*n* = 875). Black squares indicate mean differences, horizontal lines through black squares indicate 95% CI, and green diamonds indicate pooled effect sizes shown using a fixed-effect Hedges model.

The researchers found that people with prediabetes and diabetes who had high levels of glycosylated hemoglobin (HbA1c) compared to normal blood sugar had significant cognitive declines over 10 years. When HbA1c is increased by 1 mmol/ml, the *Z*-score of global cognition (−0.0009 SD/year), the *Z*-score of memory (−0.0005 SD/year), and the *Z*-score of executive function (−0.0008 SD/year) all decrease [27]. The study found a linear correlation between cognitive dysfunction and glycosylated hemoglobin levels. Compared with prediabetic patients, with the increase in HbA1c level, the cognitive dysfunction of diabetic patients declined more significantly.  Figure 5 illustrates the efficacy of DPP4 inhibitors on HBA1c. A fixed-effect model was used in 434 subjects in the experimental group and 441 in the control group. After standardization, compared with the control group, the HBA1c of the experimental group decreased by 0.34 (95% CI [−0.48, −0.21]), and the *I*^2^ value was 89% (*P* < 0.05), indicating that DPP4 inhibitor can effectively reduce HBA1c, so as to improve the cognitive impairment of diabetic patients.

### 3.7. Relationship between DPP4i and Blood Glucose at 2 hours after Meal

**Figure 6:**
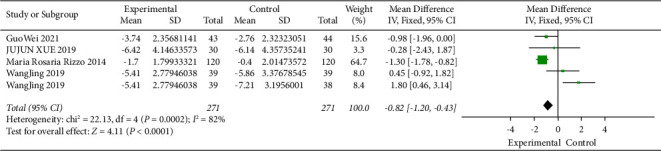
Meta-analysis of the impact of DPP4i on 2-hour postprandial glucose. Forest map showing the difference in 2-hour postprandial glucose variations with diabetes treated or not treated with DPP4i in five trials (*n* = 542). Black squares indicate mean differences, horizontal lines through black squares indicate 95% CI, and green diamonds indicate pooled effect sizes shown using a fixed-effect Hedges model.

Figure 6 illustrates the effect of DPP4 inhibitors on blood glucose at 2 hours after a meal. A fixed-effect model was used in 271 subjects in the experimental group and 271 in the control group. Post-standardisation, blood glucose at 2-hour postprandial was reduced by 0.82 (95% CI [−1.2, −0.43]), and *I*^2^ value was 82% (*P* < 0.05), which was statistically significant, indicating that the DPP4 inhibitor could effectively reduce blood glucose at 2-hour postprandial.

### 3.8. Subgroup Analysis

The effect of taking the same drug will naturally differ with the age, nationality, course of treatment, and dosage of different users. Subgroup analysis explores possible causes on the basis of two perspectives: treatment time and age differences among drug users.

#### 3.8.1. Cognitive Score

The overall cognitive score increased with DPP4i compared to the control group 0.99 (95% [0.59, 1.38]), and the difference is significant (*P* < 0.05), suggesting that cognitive impairment was improved in all patients treated with DPP4i regardless of the duration of treatment or age difference, and people aged 60 to 70 years had better treatment effects at 0–180 days. Moreover, meta-analysis showed that the heterogeneity of treatment course subgroup analysis was not reduced in the two groups. Therefore, the duration of the treatment course could not explain the heterogeneity between the studies. In the age subgroup analysis, only one of the two groups showed a decrease in heterogeneity, so the age difference could not explain the interstudy heterogeneity (Figures S1 and S2).

#### 3.8.2. Fasting Blood Glucose

Overall fasting glycemic index decreased with DPP4i compared to the control group 0.52 (95% CI [−0.68, −0.37]), and the difference is significant. Subgroup analysis showed that there was a significant difference in treatment effect by difference of age, but no significant difference between different durations of treatment course. Meta-analysis showed no reduction in the heterogeneity of duration of treatment course and age subgroup analyses in either group (Figures S3 and S4).

#### 3.8.3. Glycosylated Hemoglobin

Overall glycosylated hemoglobin decreased with DPP4i compared to the control group by 0.34 (95% CI [−0.48, −0.21]), and the difference is significant. Subgroup analysis showed that, overall, there were statistically significant differences in treatment outcomes by age and course of treatment, and meta-analysis showed that heterogeneity was not reduced among treatment groups, so different durations of treatment could not explain the heterogeneity between the two studies. However, the reduced heterogeneity found in different age groups is likely to be the reason for the high heterogeneity of the study results, which needs to be carefully understood (Figures S5 and S6).

#### 3.8.4. Blood Glucose at 2 hours after Meal

Overall blood glucose at 2 hours after meal decreased with DPP4i compared to the control group 0.82 (95% CI [−1.2, −0.43]), and the difference is significant. Subgroup analysis showed that there were statistical differences among different treatment courses. The meta-analysis showed that there was still high heterogeneity between the two groups of patients in different courses of treatment (Figure S7).

## 4. Discussion

Diabetes is a chronic disease that endangers the normal function of the human body. It can be divided into type 1 diabetes mellitus and T2D according to different pathological mechanisms. DPP-4 is a multifunctional serine protease that regulates immune cell-mediated *β* cell destruction and immune cell function, thereby prolonging the progression of type I diabetes mellitus. Both type I and T2D carry the risk of cognitive impairment. However, the negative impact of diabetes on cognition in older patients is greater in T2D than in type I diabetes. It has been reported [28] that DPP4i has neuroprotective effects and can lead to the improvement of cognitive and noncognitive dysfunctions of the nervous system. However, there are few studies on the cognitive dysfunction of DPP4i in type I diabetes, so we do not do further studies. T2D is related to the development of cognitive impairment. Some scholars believe that the mechanism of cognitive impairment with T2D may be inflammation, NOS, oxidative stress, and influence on blood vessels passing through the brain [29]. Now, studies have shown the potential neuroprotective effect of DPP4i, which have been shown to reverse amyloid deposition in cognitive impairment in Alzheimer's disease [30]. However, there is still disagreement about the cognitive effect of T2D.

In our meta-analysis, DPP4 inhibitors showed an improved effect on cognitive impairment in T2D, and DPP4i has previously been shown to have an improved effect on cognitive impairment [31]. Of course, broader cognitive tests are needed to support this idea. There are several mechanisms to explain this effect. DPP4i is a class of oral hypoglycemic agents whose function is to prevent gastrointestinal degradation of glucagon-like peptide-1 (GLP-1) and glucose-dependent insulin polypeptide, thereby improving blood sugar [32]. GLP-1 has been shown to act as a neurotrophic factor and prevent neurodegeneration, possibly by facilitating long-term enhancement, raising neurite growth, and promoting synaptic formation in a manner similar to nerve growth factor [33]. It has a potential protective effect on cognitive impairment [34]. In addition, fasting blood glucose, 2-hour postprandial blood glucose, and glycosylated hemoglobin were associated with cognitive impairment, and cognitive dysfunction was found to be linearly correlated with the levels of these factors. Our data also proved that these three variables were well improved under the action of DPP4i.

More research is needed to determine whether the type of drug used in the control group had an effect on the results. Previous studies [35] have shown that metformin users have a lower risk of dementia compared to sulfonylureas users, and the researches support the hypothesis that metformin provides more neuroprotection for dementia than sulfonylureas, but further work is needed to assess causality. However, no studies have proved that DPP4i is more effective than metformin in improving the cognitive impairment of diabetic patients. Only Zheng et al.'s study [36] showed that the use of metformin was associated with better memory performance over time, while the use of DPP4i was associated with a slower rate of memory decline. The interaction effect showed that the benefit of the DPP4 inhibitor in the APOE epsilon 4 vector was greater. Therefore, our research is more significant.

In addition, previous studies [37] have proved that the level of blood glucose and hemoglobin are related to the risk of dementia in the elderly. We considered whether the cognitive improvement after DPP4 inhibitor treatment was caused by the drug itself or by lowering blood glucose and haemoglobin. According to previous studies [38], brain-derived neurotrophic factor (BDNF) decreases and DPP4 activity increases in the peripheral circulation of mild cognitive impairment, and the negative correlation is caused by oxidative stress and inflammation. In addition, MCI patients with normal glucose tolerance also have lower peripheral circulation activity of BDNF and increased DPP4, and the use of DPP4 inhibitors can improve cognitive dysfunction. Therefore, we believe that it is the drug action of the DPP4 inhibitor itself that leads to the cognitive improvement. Further research is needed.

Finally, the effect of taking the same drug will naturally vary with the age, nationality, course of treatment, and dosage of different users. We hypothesized that the high heterogeneity of the effects of DPP4 inhibitors on cognitive impairment was due to other factors such as country, age, sex, and duration of treatment. Finally, we used subgroup analysis to reduce heterogeneity. The main analysis included cognitive score, fasting blood glucose, glycosylated hemoglobin, and two hours postprandial blood glucose, and the variable factors were different in treatment duration and age. However, these subgroup analyses also had moderate and high heterogeneity, and only the subgroup analysis of glycosylated hemoglobin did heterogeneity decrease across age groups. In addition, subgroup analyses based on cognitive scores were conducted, and DPP4i has better results in patients aged 60 to 70 years old. At the same time, the treatment time of 0 to 180 days is better, which indicates the importance of age and time of medication in clinical studies.

All of these suggest that DPP4 inhibitors have a positive effect on cognitive impairment in T2D and have a potential role in preventing cognitive impairment. The significance of this review and meta-analysis is to give further recommendations for clinical research, which will have positive implications for the protection of cognitive dysfunction in diabetic patients.

### 4.1. Limitations

Limitations are as follows: (1) cognitive impairment changes with age, and the prognostic value of DPP4 inhibitors also has certain risks. Although the data we collected were the result of multivariate adjustment, other confounding factors could not be excluded. (2) Some differences can be observed in the inclusion of RCTS, and there are random errors. (3) There was significant heterogeneity in the study, such as age, experimental design, drug dosage, and other factors, but due to limited data, we could not do subgroup analysis to test the heterogeneity. (4) This study is mainly to prove the effect of DPP4 hypoglycemic agents on diabetic patients with cognitive impairment, which should be compared with other hypoglycemic agents. However, due to the lack of data such as dosage and use time in literature, more studies are needed to solve the problem. (5) The patients in our paper are from Asia, America, and Europe. There is a lack of research in other regions, and more data are needed to confirm the differences in drug efficacy among different ethnic groups.

## 5. Conclusion

Based on the available data discussed, our data suggest that DPP4 inhibitors remarkably improve cognitive dysfunction in patients with T2D and decline fasting blood glucose, 2-hour postprandial blood glucose, and glycosylated hemoglobin. Subgroup analysis showed that people aged 60 to 70 years had better treatment effects at 0–180 days. However, more precise conclusions need a larger data size and sample size, which, we hope, can be solved in the future.

## Data Availability

The data that support the findings of this study are available upon request from the corresponding author.

## Supplementary Materials

Supplementary Materials (1) PRISMA Checklist: reporting standards for this systematic review and meta-analysis. (2) Table 1: characteristics of the ten prospective studies. (3) Figure S1: subgroup analysis of the effect of different treatment courses on cognitive impairment. (4) Figure S2: subgroup analysis of the effects of different age groups on cognitive impairment was analyzed. (5) Figure S3: subgroup analysis of the effect of different treatment durations on fasting blood glucose was analyzed. (6) Figure S4: subgroup analysis of the effect of different age groups on fasting blood glucose was analyzed. (7) Figure S5: subgroup analysis of the effect of different treatment durations on glycosylated hemoglobin was analyzed. (8) Figure S6: subgroup analysis of the effect of different age groups on glycosylated hemoglobin was analyzed. (9) Figure S7: subgroup analysis of the effect of different treatment durations on blood glucose at 2 hours after meal was analyzed.

## References

[1] Groeneveld O. N., Kappelle L. J., Biessels G. J. (2016). Potentials of incretin-based therapies in dementia and stroke in Type 2 diabetes mellitus mellitus. *Journal of Diabetes Investigation*.

[2] Cukierman T., Gerstein H. C., Williamson J. D. (2005). Cognitive decline and dementia in diabetes--systematic overview of prospective observational studies. *Diabetologia*.

[3] Roberts R. O., Knopman D. S., Geda Y. E. (2014). Association of diabetes with amnestic and nonamnestic mild cognitive impairment. *Alzheimer’s and Dementia*.

[4] Chatterjee S., Peters S. A., Woodward M. (2016). Type 2 diabetes as a risk factor for dementia in women compared with men: a pooled analysis of 2.3 million people comprising more than 100,000 cases of dementia. *Diabetes Care*.

[5] Zheng T., Qin L., Chen B. (2016). Association of plasma DPP4 activity with mild cognitive impairment in elderly patients with type 2 diabetes: results from the GDMD study in China. *Diabetes Care*.

[6] Xie Y., Zhou Q., He Q., Wang X., Wang J. (2023). Opportunities and challenges of incretin-based hypoglycemic agents treating type 2 diabetes mellitus from the perspective of physiological disposition. *Acta Pharmaceutica Sinica B*.

[7] Salcedo I., Tweedie D., Li Y., Greig N. H. (2012). Neuroprotective and neurotrophic actions of glucagon-like peptide-1: an emerging opportunity to treat neurodegenerative and cerebrovascular disorders. *British Journal of Pharmacology*.

[8] Zhong J., Rao X., Rajagopalan S. (2013). An emerging role of dipeptidyl peptidase 4 (DPP4) beyond glucose control: potential implications in cardiovasculardisease. *Atherosclerosis*.

[9] Zhong J., Gong Q., Goud A., Srinivasamaharaj S., Rajagopalan S. (2015). Recent advances in dipeptidyl-peptidase-4 inhibition therapy: lessons from the bench and clinical trials. *Journal of Diabetes Research*.

[10] Ma N., Liang Y., Yue L., Liu P., Xu Y., Zhu C. (2022). The identities of insulin signaling pathway are affected by overexpression of Tau and its phosphorylation form. *Frontiers in Aging Neuroscience*.

[11] Zheng J., Wang Y., Liu Y. (2022). cPKC*γ* deficiency exacerbates autophagy impairment and hyperphosphorylated tau buildup through the AMPK/mTOR pathway in mice with type 1 diabetes mellitus. *Neuroscience Bulletin*.

[12] Wang Y. Y., Yan Q., Huang Z. T. (2021). Ameliorating ribosylation-induced amyloid-*β* pathology by berberine via inhibiting mTOR/p70S6K signaling. *Journal of Alzheimer’s Disease*.

[13] Biessels G. J., Verhagen C., Janssen J. (2021). Effects of linagliptin vs glimepiride on cognitive performance in Type 2 diabetes mellitus: results of the randomised double-blind, active-controlled CAROLINA-COGNITION study. *Diabetologia*.

[14] Biessels G. J., Verhagen C., Janssen J. (2019). Effect of linagliptin on cognitive performance in patients with type 2 diabetes mellitus and cardiorenal comorbidities: the CARMELINA randomized trial. *Diabetes Care*.

[15] Higgins J. P., Altman D. G., Gøtzsche P. C. (2011). The Cochrane Collaboration’s tool for assessing risk of bias in randomised trials. *BMJ*.

[16] Alagiakrishnan K., Zhao N., Mereu L., Senior P., Senthilselvan A. (2013). Montreal Cognitive Assessment is superior to Standardized Mini-Mental Status Exam in detecting mild cognitive impairment in the middle-aged and elderly patients with Type 2 diabetes mellitus mellitus. *BioMed Research International*.

[17] Li Y., Liu Z. (2018). Network Meta-analysis of tonifying traditional Chinese medicine injections in the adjuvant. *Treatment for Viral Myocarditis*.

[18] Ates Bulut E., Sahin Alak Z. Y., Dokuzlar O. (2019). Cognitive and metabolic outcomes of vildagliptin addition to the therapy in patients with Type 2 diabetes mellitus mellitus: 26 week follow-up study. *Archives of Gerontology and Geriatrics*.

[19] Xue J., Wang C., Pan C. (2019). Effect of DPP-4 inhibitor on elderly patients with T2DM combined with MCI. *Experimental and Therapeutic Medicine*.

[20] Jin L. (2017). Effects of DPP-4 inhibitor for the cognitive dysfunction of elderly patients with Type 2 diabetes mellitus mellitus and Alzheimer disease. *China Medical Herald*.

[21] Borzì A. M., Condorelli G., Biondi A. (2019). Effects of vildagliptin, a DPP-4 inhibitor, in elderly diabetic patients with mild cognitive impairment. *Archives of Gerontology and Geriatrics*.

[22] Rizzo M. R., Barbieri M., Boccardi V., Angellotti E., Marfella R., Paolisso G. (2014). Dipeptidyl peptidase-4 inhibitors have protective effect on cognitive impairment in aged diabetic patients with mild cognitive impairment. *The Journals of Gerontology Series A: Biological Sciences and Medical Sciences*.

[23] Wang J., Liu P., Dong S., Feng H., Zhou H. (2019). Efficacy and safety of alogliptin for patients with type 2 diabetic mild cognitive impairment. *Journal of Hebei Medical University*.

[24] Guo W., Li W., Peng C., Xie H. (2021). Effect of sitagliptin on type 2 diabetic patients with mild cognitive impairment. *Medical Journal of Wuhan University*.

[25] Hu Z., Zhang H., Wang Y., Xia W. (2018). Effect and its mechanism of Linagliptin on mild cognitive impairment in elderly Type 2 diabetes mellitus mellitus patients. *Chinese General Practice*.

[26] Wang K., Xu L., Liu L., Zhan S., Wang S., Song Y. (2022). Sex differences in the association between the change in triglyceride-glucose index and cognitive decline: a population-based cohort study. *Journal of Affective Disorders*.

[27] Zheng F., Yan L., Yang Z., Zhong B., Xie W. (2018). HbA(1c), diabetes and cognitive decline: the English longitudinal study of ageing. *Diabetologia*.

[28] Meier J. J. (2012). GLP-1 receptor agonists for individualized treatment of type 2 diabetes mellitus. *Nature Reviews Endocrinology*.

[29] Alkasabera A., Onyali C. B., Anim-Koranteng C. (2021). The effect of type-2 diabetes on cognitive status and the role of anti-diabetes medications. *Cureus*.

[30] Agarwal P., Alok S., Fatima A., Singh P. P. (2013). Herbal remedies for neurodegenerative disorder (Alzheimer’s disease): a Review. *International Journal of Pharma Sciences and Research*.

[31] Xiao L., Ge B., Chen X. (2019). The relationship between plasma DPP4 activity to BDNF ratio and mild cognitive impairment in elderly population with normal glucose tolerance. *Frontiers in Aging Neuroscience*.

[32] Mossello E., Ballini E., Boncinelli M. (2011). Glucagon-like peptide-1, diabetes, and cognitive decline: possible pathophysiological links and therapeutic opportunities. *Experimental Diabetes Research*.

[33] Shannon R. P. (2013). DPP-4 inhibition and neuroprotection: do mechanisms matter?. *Diabetes*.

[34] Schnapp G., Klein T., Hoevels Y., Bakker R. A., Nar H. (2016). Comparative analysis of binding kinetics and thermodynamics of dipeptidyl peptidase-4 inhibitors and their relationship to structure. *Journal of Medicinal Chemistry*.

[35] Song J., Bai H., Xu H., Xing Y., Chen S. (2022). HbA1c variability and the risk of dementia in patients with diabetes: a meta-analysis. *International Journal of Clinical Practice*.

[36] Zheng T., Qin L., Chen B. (2016). Association of plasma DPP4 activity with mild cognitive impairment in elderly patients with type 2 diabetes: results from the GDMD study in China. *Diabetes Care*.

[37] Wang P., Li Y., Wang M. (2024). Comparing glycemic traits in defining diabetes among rural Chinese older adults. *PLoS One*.

[38] Al-Rawaf H. A., Alghadir A. H., Gabr S. A. (2021). Molecular changes in circulating microRNAs’ expression and oxidative stress in adults with mild cognitive impairment: a biochemical and molecular study. *Clinical Interventions in Aging*.

