# Supplementary material for: Effects of DPP4 Inhibitors as Neuroprotective Drug on Cognitive Impairment in Patients with Type 2 Diabetes Mellitus: A Meta-Analysis and Systematic Review
Source: Int J Endocrinol. 2024 Feb 13;2024:9294113. doi: 10.1155/2024/9294113 (PMC10878760; doi:10.1155/2024/9294113)
Supplement: Supplementary Materials — (1) PRISMA Checklist: reporting standards for this systematic review and meta-analysis. (2) Table 1: characteristics of the ten prospective studies. (3) Figure S1: subgroup analysis of the effect of different treatment courses on cognitive impairment. (4) Figure S2: subgroup analysis of the effects of different age groups on cognitive impairment was analyzed. (5) Figure S3: subgroup analysis of the effect of different treatment durations on fasting blood glucose was analyzed. (6) Figure S4: subgroup analysis of the effect of different age groups on fasting blood glucose was analyzed. (7) Figure S5: subgroup analysis of the effect of different treatment durations on glycosylated hemoglobin was analyzed. (8) Figure S6: subgroup analysis of the effect of different age groups on glycosylated hemoglobin was analyzed. (9) Figure S7: subgroup analysis of the effect of different treatment durations on blood glucose at 2 hours after meal was analyzed. [file 9294113.f1.zip › Supplementary pictures (1).docx]

**Supplementary pictures**


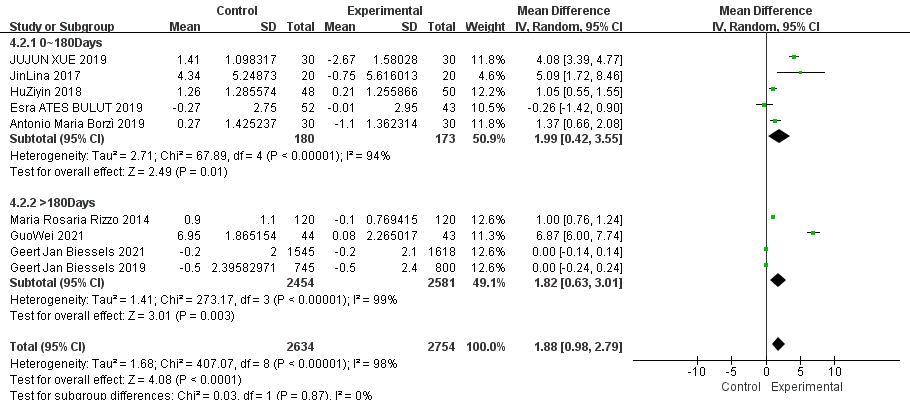


Figure S1 The effect of different treatment courses on cognitive impairment.The forest map showed the difference in cognitive impairment among adults with diabetes who received or did not receive DPP4i (n=5388). The mean difference (black square), 95% CI (horizontal line through black square), and merge effect size (green diamond) were represented using the random effects Hedges model.


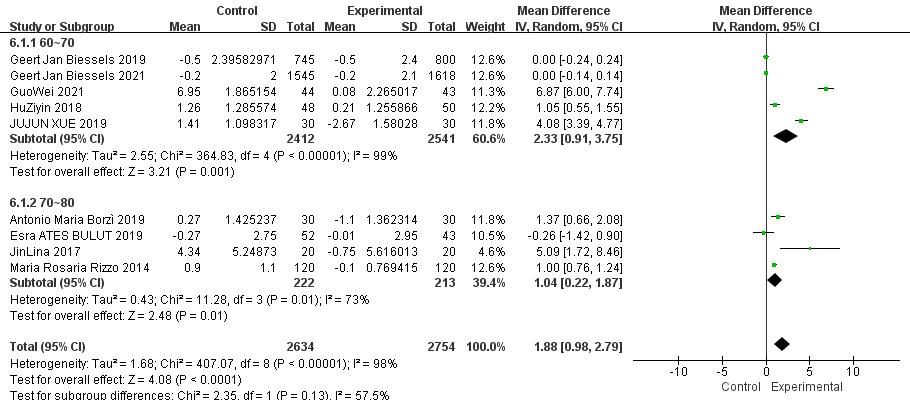


Figure S2 The effects of different age groups on cognitive impairment were analyzed. Forest maps show the difference in cognitive impairment between adults with diabetes who received DPP4i and those who did not (n=5388). Mean difference (black square), 95% CI (horizontal line through black square), and merge effect size (green diamond) were represented using the random effects Hedges model.


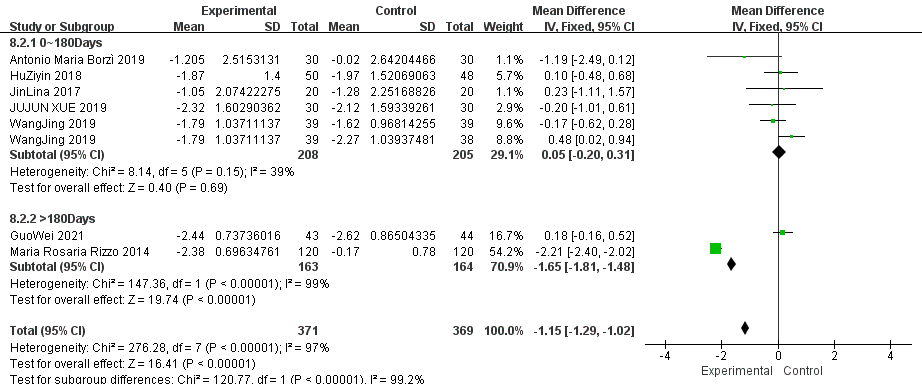


Figure S3 Effect of different treatment duration on fasting blood glucose were analyzed. Forest maps show the difference in fasting blood glucose between adults with diabetes who received DPP4i and those who did not (n=740). Mean difference (black square), 95% CI (horizontal line through black square), and merge effect size (green diamond) were represented using the fixed-effect Hedges models.


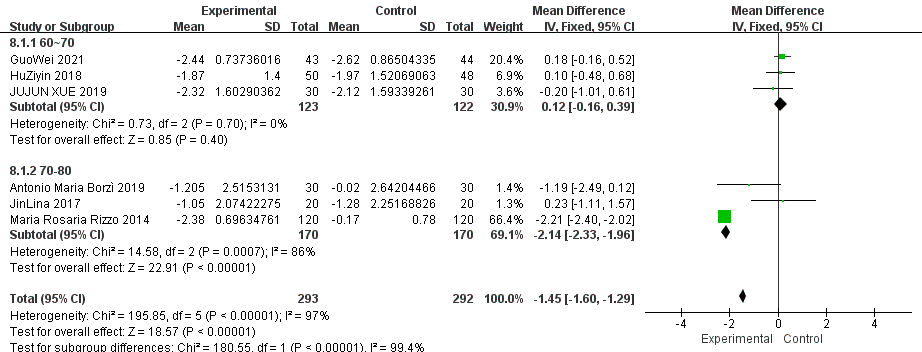


Figure S4 Effect of different age groups on fasting blood glucose were analyzed. Forest maps show the difference in fasting blood glucose between adults with diabetes who received DPP4i and those who did not (n=585). Mean difference (black square), 95% CI (horizontal line through black square), and merge effect size (green diamond) were represented using the fixed-effect Hedges models.


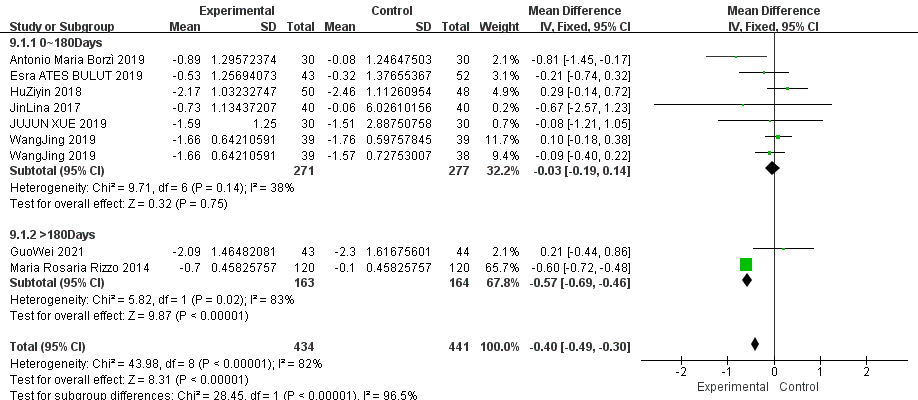


Figure S5 Effect of different treatment duration on glycosylated hemoglobin were analyzed. Forest maps show the difference in fasting blood glucose between adults with diabetes who received DPP4i and those who did not (n=875). Mean difference (black square), 95% CI (horizontal line through black square), and merge effect size (green diamond) were represented using the fixed-effect Hedges models.


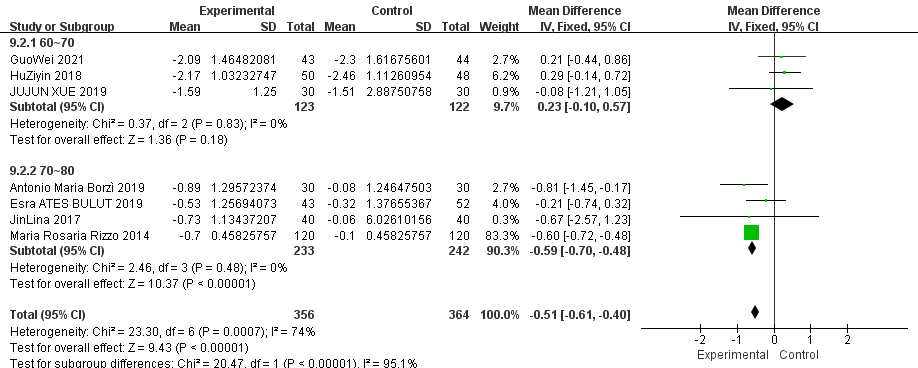


Figure S6 Effect of different age groups on glycosylated hemoglobin were analyzed. Forest maps show the difference in fasting blood glucose between adults with diabetes who received DPP4i and those who did not (n=720). Mean difference (black square), 95% CI (horizontal line through black square), and merge effect size (green diamond) were represented using the fixed-effect Hedges models.


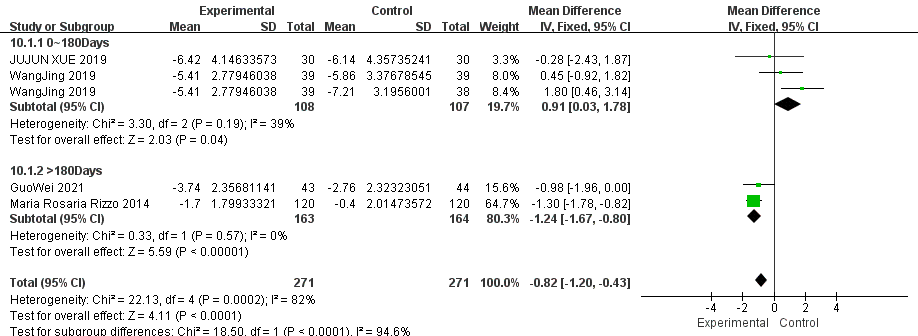


Figure S7 Effect of different treatment duration on blood glucose at 2 hours after meal were analyzed. Forest maps show the difference in fasting blood glucose between adults with diabetes who received DPP4i and those who did not (n=542). Mean difference (black square), 95% CI (horizontal line through black square), and merge effect size (green diamond) were represented using the fixed-effect Hedges models.
