# Supplementary material for: Effects of DPP4 Inhibitors as Neuroprotective Drug on Cognitive Impairment in Patients with Type 2 Diabetes Mellitus: A Meta-Analysis and Systematic Review
Source: Int J Endocrinol. 2024 Feb 13;2024:9294113. doi: 10.1155/2024/9294113 (PMC10878760; doi:10.1155/2024/9294113)
Supplement: Supplementary Materials — (1) PRISMA Checklist: reporting standards for this systematic review and meta-analysis. (2) Table 1: characteristics of the ten prospective studies. (3) Figure S1: subgroup analysis of the effect of different treatment courses on cognitive impairment. (4) Figure S2: subgroup analysis of the effects of different age groups on cognitive impairment was analyzed. (5) Figure S3: subgroup analysis of the effect of different treatment durations on fasting blood glucose was analyzed. (6) Figure S4: subgroup analysis of the effect of different age groups on fasting blood glucose was analyzed. (7) Figure S5: subgroup analysis of the effect of different treatment durations on glycosylated hemoglobin was analyzed. (8) Figure S6: subgroup analysis of the effect of different age groups on glycosylated hemoglobin was analyzed. (9) Figure S7: subgroup analysis of the effect of different treatment durations on blood glucose at 2 hours after meal was analyzed. [file 9294113.f1.zip › Table 1 Characteristics of the ten prospective studies.pdf]

Table 1 Characteristics of the ten prospective studies

Author	Year	Research design	Treatment duration	Sample number	Sex ratio (female)	Age	Control	Adjustment variable
Geert Jan Biessels et al.,[16]	2021	RCT	160Weeks	3163	1203（3163）	64.4±9.1	sulfonylureas	BMI、Course of disease、HbA1c、FBG、LDL
Esra ATES BULUT et al.,[17]	2019	RCT	180Days	95	47.5（95）	74.4±7.9	placebo	BMI、Course of disease、HbA1c、LDL
JUJUN XUE et al.,[18]	2019	RCT	180Days	60	29（60）	68.5±7.1	sulfonylureas	BMI、Course of disease、HbA1c、FBG、LDL、CRP
Geert Jan Biessels et al.,[19]	2019	RCT	2.5Years	1545	541（1545）	67.8±8.3	placebo	BMI、Course of disease、LDL
JinLina et al.,[20]	2017	RCT	180Days	80	39（80）	72.57±8.3	sulfonylureas	BMI、Course of disease、HbA1c、FBG
Antonio Maria Borzì et al.,[21]	2019	RCT	180Days	60	33（60）	77.67±8.23	metformin	BMI、Course of disease、HbA1c、FBG、LDL
Maria Rosaria Rizzo et al.,[22]	2014	RCT	2Years	240	146（240）	73.1±4.2	metformin	BMI、Course of disease、HbA1c、FBG
WangJing et al.,[23]	2019	RCT	24Weeks	78	35（78）	48.2±8.63	Alpha-glycosidase inhibitors	BMI、Course of disease、HbA1c、FBG
GuoWei et al.,[24]	2021	RCT	48Weeks	87	43（44）	66.2±5.9	sulfonylureas	BMI、Course of disease、HbA1c、FBG
HuZiyin et al.,[25]	2018	RCT	24Weeks	98	47（98）	66.8±4.2	metformin	BMI、Course of disease、HbA1c、FBG
