# Supplementary material for: Effects of DPP4 Inhibitors as Neuroprotective Drug on Cognitive Impairment in Patients with Type 2 Diabetes Mellitus: A Meta-Analysis and Systematic Review
Source: Int J Endocrinol. 2024 Feb 13;2024:9294113. doi: 10.1155/2024/9294113 (PMC10878760; doi:10.1155/2024/9294113)
Supplement: Supplementary Materials — (1) PRISMA Checklist: reporting standards for this systematic review and meta-analysis. (2) Table 1: characteristics of the ten prospective studies. (3) Figure S1: subgroup analysis of the effect of different treatment courses on cognitive impairment. (4) Figure S2: subgroup analysis of the effects of different age groups on cognitive impairment was analyzed. (5) Figure S3: subgroup analysis of the effect of different treatment durations on fasting blood glucose was analyzed. (6) Figure S4: subgroup analysis of the effect of different age groups on fasting blood glucose was analyzed. (7) Figure S5: subgroup analysis of the effect of different treatment durations on glycosylated hemoglobin was analyzed. (8) Figure S6: subgroup analysis of the effect of different age groups on glycosylated hemoglobin was analyzed. (9) Figure S7: subgroup analysis of the effect of different treatment durations on blood glucose at 2 hours after meal was analyzed. [file 9294113.f1.zip › PRISMA Checklist (1).doc]

| **Section/topic** | **#** | **Checklist item** | **Reported on page #** |
| --- | --- | --- | --- |
| **TITLE** | | |  |
| Title | 1 | Effects of DPP4 inhibitors as neuroprotective drug on cognitive impairment in patients with Type 2 diabetes mellitus: a meta-analysis and systematic review |  |
| **ABSTRACT** | | |  |
| Structured summary | 2 | Background:Type 2 diabetes mellitus is a risk factor for cognitive impairment. DPP4 inhibitors are effective drugs for the treatment of Type 2 diabetes mellitus. But the relationship between DPP4 inhibitors and cognitive dysfunction remains unclear.  Objectives:Therefore, we used a meta-analysis to determine the association between DPP4 inhibitors and cognitive impairment in Type 2 diabetes mellitus.  Data sources:We systematically searched PubMed, CNKI, and the Cochrane Library at the time of establishment -- 2022, then made inclusion criteria and screened strategies to identify studies with more precise correlations.  Participants:Literature search and research two researchers (Yuting Yuan and Qingjie Chen) independently searched four databases combining medical keywords (Mesh) terms and keywords.  Interventions:DPP4i  Study appraisal and synthesis methods:We compared the baseline data before and after medication, extracted the Mean, standard deviation and sample size (N) from the literature, and calculated the mean (Mean) and standard deviation (SD) values difference between baseline and endpoint. Considering the differences in experimental design and measurement units, standard mean difference (SMD) was used to eliminate the difference in data.  Results:DPP4 inhibitors significantly improved cognitive dysfunction in patients with Type 2 diabetes mellitus and reduced fasting blood glucose, 2-hour postprandial blood glucose, and glycosylated hemoglobin. Subgroup analysis showed that people aged 60 to 70 years had better treatment effects at 0-180 days.  Limitations: 1) Cognitive impairment changes with age, and the prognostic value of DPP4 inhibitors also has certain risks. Although the data we collected were the result of multivariate adjustment, other confounding factors could not be excluded. 2) Some differences can be observed in the inclusion of RCTS, and there are random errors. 3) There was significant heterogeneity in the study, such as age, experimental design, drug dosage and other factors, but due to limited data, we could not do subgroup analysis to test the heterogeneity. 4) This study is mainly to prove the effect of DPP4 hypoglycemic agents on diabetic patients with cognitive impairment, which should be compared with other hypoglycemic agents. However, due to the lack of data such as dosage and use time in literature, more studies are needed to solve the problem. 5) The patients in our paper are from Asia, America and Europe. There is a lack of research in other regions, and more data is needed to confirm the differences in drug efficacy among different ethnic groups.  conclusions and implications of key findings:Based on the available data discussed, our dates suggest that DPP4 inhibitors significantly improve cognitive dysfunction in patients with Type 2 diabetes mellitus and reduce fasting blood glucose, 2-hour postprandial blood glucose, and glycosylated hemoglobin. Subgroup analysis showed that people aged 60 to 70 years had better treatment effects at 0-180 days. However, more precise conclusions need larger data size and sample size, which we hope it can be solved in future.  Systematic review registration number:CRD42023399473.[*PROSPERO (york.ac.uk)*](https://www.crd.york.ac.uk/PROSPERO/) |  |
| **INTRODUCTION** | | |  |
| Rationale | 3 | In previous studies, increased activity of dipeptidyl peptidase-4 inhibitors (DPP4) was independently associated with mild cognitive impairment (MCI) in elderly patients with Type 2 diabetes mellitus. Possibly in its effect on some risk factors associated with MCI, so it may be a therapeutic target for MCI. However, the relationship between DPP4 inhibitors and cognitive dysfunction in T2D patients remains controversial. As an oral hypoglycemic agent for T2DM, DPP-4, an intestinal glucagon-like peptide-1 (GLP-1) degrading enzyme, it can block DPP-4 to reduce GLP-1 degradation and prolong its GLP-1 effect when used. And lead to insulin activity, thus reducing blood sugar . In addition, GLP-1 has been shown to act as a neurotrophic factor and prevent neurodegeneration, possibly through long-term enhancement, enhancing neurite growth and promoting synaptic formation in a way similar to nerve growth factor . DPP4 inhibitors can effectively prevent the degradation of GLP-1 and gastric inhibitory peptide. Therefore prolonging the half-life of these incretin and prolonging the life of neurons . Such mechanisms include activation of mTOR pathway and tau hyperphosphorylation, thus inhibiting liver gluconeogenesis, reducing insulin resistance, increasing insulin sensitivity, and inhibiting inflammation. |  |
| Objectives | 4 | Participants:Patients must conform to the definition of diabetes and have a positive pathological examination or OGTT test (fasting blood glucose ≥7.0mmol/l or blood glucose ≥11.1mmol/l 2 hours after meal). Patients must have cognitive impairment and have the same cognitive scoring tools between the experimental group and the control group  Interventions:The drug in the experimental group must be DPP4 inhibitor, and the drug dose in the experimental group must be greater than the lowest effective dose.  Comparisons:Control group and DPP4i group.  Outcomes:DPP4 inhibitors significantly improved cognitive dysfunction in patients with Type 2 diabetes mellitus and reduced fasting blood glucose, 2-hour postprandial blood glucose, and glycosylated hemoglobin. Subgroup analysis showed that people aged 60 to 70 years had better treatment effects at 0-180 days.  Study design (PICOS):Basic information about relevant literature, baseline information and experimental results will be extracted, including article title, author details, journal publication year, study time, study population, duration of follow-up, baseline age, baseline weight, gender distribution, diabetes course, endpoint definition and determination of Montreal cognitive assessment score, and study results. Odds ratios and 95% confidence intervals (CI) will be calculated to assess the association between DPP-4 inhibitors and cognitive impairment. In general, we compared the baseline data before and after medication, extracted the Mean, standard deviation and sample size (N) from the literature, and calculated the mean (Mean) and standard deviation (SD) values difference between baseline and endpoint. Considering the differences in experimental design and measurement units, standard mean difference (SMD) was used to eliminate the difference in data _._ |  |
| **METHODS** | | |  |
| Protocol and registration | 5 | Systematic review registration number:CRD42023399473.[*PROSPERO (york.ac.uk)*](https://www.crd.york.ac.uk/PROSPERO/) |  |
| Eligibility criteria | 6 | Specify study characteristics：  Participants:Patients must conform to the definition of diabetes and have a positive pathological examination or OGTT test (fasting blood glucose ≥7.0mmol/l or blood glucose ≥11.1mmol/l 2 hours after meal). Patients must have cognitive impairment and have the same cognitive scoring tools between the experimental group and the control group.  Interventions:The drug in the experimental group must be DPP4 inhibitor, and the drug dose in the experimental group must be greater than the lowest effective dose.  Comparisons:Control group and DPP4i group.  Outcomes: DPP4 inhibitors significantly improved cognitive dysfunction in patients with Type 2 diabetes mellitus and reduced fasting blood glucose, 2-hour postprandial blood glucose, and glycosylated hemoglobin. Subgroup analysis showed that people aged 60 to 70 years had better treatment effects at 0-180 days.  Study design: Basic information about relevant literature, baseline information and experimental results will be extracted, including article title, author details, journal publication year, study time, study population, duration of follow-up, baseline age, baseline weight, gender distribution, diabetes course, endpoint definition and determination of Montreal cognitive assessment score, and study results. Odds ratios and 95% confidence intervals (CI) will be calculated to assess the association between DPP-4 inhibitors and cognitive impairment. In general, we compared the baseline data before and after medication, extracted the Mean, standard deviation and sample size (N) from the literature, and calculated the mean (Mean) and standard deviation (SD) values difference between baseline and endpoint. Considering the differences in experimental design and measurement units, standard mean difference (SMD) was used to eliminate the difference in data_._  Report characteristics：  Years considered: PubMed, CNKI, and the Cochrane Library at the time of establishment -- 2022.  Language: English  Publication status: All included articles have been published. |  |
| Information sources | 7 | We systematically searched PubMed, CNKI, and the Cochrane Library at the time of establishment -- 2022 |  |
| Search | 8 | Cochrane library trials matching diabetes in Title Abstract Keyword AND DPP-4 Inhibitor or Dipeptidyl Peptidase IV Inhibitors or DPP-IV Inhibitors in Title Abstract Keyword AND Cognitive Dysfunctions or Cognitive Impairments or Mental Deterioration in Title Abstract Keyword - (Word variations have been searched) |  |
| Study selection | 9 | Inclusion and exclusion criteria were set according to PICOS (Population, Intervention, Comparison, Outcomes, and Study Design).  The inclusion criteria are as follows:  1) Published reports on the effects of DPP4 inhibitors (Sitagliptin, Vigliptin, Ligliptin, Alogliptin, Saxagliptin) on people with diabetic cognitive impairment  2) The study design must be an RCTS with placebo or parallel controls  3) Patients must conform to the definition of diabetes and have a positive pathological examination or OGTT test (fasting blood glucose ≥7.0mmol/l or blood glucose ≥11.1mmol/l 2 hours after meal).  4) Patients must have cognitive impairment and have the same cognitive scoring tools between the experimental group and the control group  5) The drug in the experimental group must be DPP4 inhibitor, and the drug dose in the experimental group must be greater than the lowest effective dose  The exclusion criteria are as follows:  1) The deviation of research data is obvious and the reliability is low  2) Patients with diabetes or cognitive impairment were not involved  3) There was no DPP4 inhibitor in the experimental group  4) Literature without data |  |
| Data collection process | 10 | Basic information about relevant literature, baseline information and experimental results will be extracted, including article title, author details, journal publication year, study time, study population, duration of follow-up, baseline age, baseline weight, gender distribution, diabetes course, endpoint definition and determination of Montreal cognitive assessment score, and study results. |  |
| Data items | 11 | Basic information about relevant literature, baseline information and experimental results will be extracted, including article title, author details, journal publication year, study time, study population, duration of follow-up, baseline age, baseline weight, gender distribution, diabetes course, endpoint definition and determination of Montreal cognitive assessment score , and study results. Odds ratios and 95% confidence intervals (CI) will be calculated to assess the association between DPP-4 inhibitors and cognitive impairment. In general, we compared the baseline data before and after medication, extracted the Mean, standard deviation and sample size (N) from the literature, and calculated the mean (Mean) and standard deviation (SD) values difference between baseline and endpoint. Considering the differences in experimental design and measurement units, standard mean difference (SMD) was used to eliminate the difference in data _._ |  |
| Risk of bias in individual studies | 12 | According to the Cochrane Handbook for Systematic Evaluation of Intervention , the quality of selected studies was evaluated in six aspects: random sequence generation, assignment hiding, participant and personnel blindness, outcome evaluation blindness, incomplete outcome data, and selective reporting. These biases will be independently assessed by 2 review authors for each included study. Among them, the definition of allocation hiding is the selective bias caused by the imperfect follow-up allocation scheme, and the definition of selective reporting is the reporting bias caused by the selective reporting results. The literature we included included one risk on the allocation of hidden risks, and six risks due to incomplete other data in the selective report. The Cochrane bias risk assessment tool will be used to mark each bias as "yes" (low bias risk), "no" (high bias risk), or "unclear" (uncertain bias risk). Any disagreement will be discussed with the third review writer. If there is any disagreement, please contact the author for request. |  |
| Summary measures | 13 | We compared the baseline data before and after medication, extracted the Mean, standard deviation and sample size (N) from the literature, and calculated the mean (Mean) and standard deviation (SD) values difference between baseline and endpoint. Considering the differences in experimental design and measurement units, standard mean difference (SMD) was used to eliminate the difference in data. |  |
| Synthesis of results | 14 | Odds ratios and 95% confidence intervals (CI) will be calculated to assess the association between DPP-4 inhibitors and cognitive impairment. |  |

Page 1 of 2

| **Section/topic** | **#** | **Checklist item** | **Reported on page #** |
| --- | --- | --- | --- |
| Risk of bias across studies | 15 | The literature we included included one risk on the allocation of hidden risks, and six risks due to incomplete other data in the selective report. The Cochrane bias risk assessment tool will be used to mark each bias as "yes" (low bias risk), "no" (high bias risk), or "unclear" (uncertain bias risk). Any disagreement will be discussed with the third review writer. If there is any disagreement, please contact the author for request. |  |
| Additional analyses | 16 | Subgroup analysis showed that people aged 60 to 70 years had better treatment effects at 0-180 days. |  |
| **RESULTS** | | |  |
| Study selection | 17 |  |  |
| Study characteristics | 18 |  |  |
| Risk of bias within studies | 19 |  |  |
| Results of individual studies | 20 |   Meta-analysis and migration analysis of the effect of DPP4i on cognitive impairment. |  |
| Synthesis of results | 21 |   Meta-analysis and migration analysis of the effect of DPP4i on cognitive impairment.    Meta-analysis and migration analysis of the effect of DPP4i on fasting blood glucose.    Meta-analysis and migration analysis of the effect of DPP4i on glycosylated hemoglobin.    Meta-analysis and migration analysis of the effect of DPP4i on blood glucose at 2 hours after meal. |  |
| Risk of bias across studies | 22 |  |  |
| Additional analysis | 23 |   Subgroup analysis of the effect of different treatment courses on cognitive impairment.The forest map showed the difference in cognitive impairment among adults with diabetes who received or did not receive DPP4i (n=5388). The mean difference (black square), 95% CI (horizontal line through black square), and merge effect size (green diamond) were represented using the random effects Hedges model. |  |
| **DISCUSSION** | | |  |
| Summary of evidence | 24 | 1、Based on the available data discussed, our dates suggest that DPP4 inhibitors significantly improve cognitive function in patients with type Ⅱ diabetes. 2、At the same time, DPP4 inhibitors reduce fasting blood glucose, 2-hour postprandial blood glucose, and glycosylated hemoglobin. 3、Subgroup analysis showed that people aged 60 to 70 years had better treatment effects at 0-180 days. |  |
| Limitations | 25 | Limitations: 1) Cognitive impairment changes with age, and the prognostic value of DPP4 inhibitors also has certain risks. Although the data we collected were the result of multivariate adjustment, other confounding factors could not be excluded. 2) Some differences can be observed in the inclusion of RCTS, and there are random errors. 3) There was significant heterogeneity in the study, such as age, experimental design, drug dosage and other factors, but due to limited data, we could not do subgroup analysis to test the heterogeneity. 4) This study is mainly to prove the effect of DPP4 hypoglycemic agents on diabetic patients with cognitive impairment, which should be compared with other hypoglycemic agents. However, due to the lack of data such as dosage and use time in literature, more studies are needed to solve the problem. 5) The patients in our paper are from Asia, America and Europe. There is a lack of research in other regions, and more data is needed to confirm the differences in drug efficacy among different ethnic groups. |  |
| Conclusions | 26 | Based on the available data discussed, our dates suggest that DPP4 inhibitors significantly improve cognitive dysfunction in patients with Type 2 diabetes mellitus and reduce fasting blood glucose, 2-hour postprandial blood glucose, and glycosylated hemoglobin. Subgroup analysis showed that people aged 60 to 70 years had better treatment effects at 0-180 days. However, more precise conclusions need larger data size and sample size, which we hope it can be solved in future. |  |
| **FUNDING** | | |  |
| Funding | 27 | This work was supported by the National Natural Science Foundation of China (82270892, 81870173, and 82073852), Development and utilization of experimental animal resources in Hubei Province (2021DFE025), Natural Science Foundation of Hubei Province (2022CFB287), Xianning City Science and Technology Plan Project (2022ZRKX052, 2021SFYF006), School projects of Hubei University of Science and Technology (2022T01, 2021WG05, 2021TNB01). |  |

*From:*  Moher D, Liberati A, Tetzlaff J, Altman DG, The PRISMA Group (2009). Preferred Reporting Items for Systematic Reviews and Meta-Analyses: The PRISMA Statement. PLoS Med 6(6): e1000097. doi:10.1371/journal.pmed1000097

For more information, visit: **www.prisma-statement.org**.

Page 2 of 2
